# The Structural Ceramics Database: Technical Foundations

**DOI:** 10.6028/jres.094.006

**Published:** 1989

**Authors:** R. G. Munro, F. Y. Hwang, C. R. Hubbard

**Affiliations:** Ceramics Division National Institute of Standards and Technology Gaithersburg, MD 20899

**Keywords:** ceramics, computerized database, material properties, Structural Ceramics Database, user-friendly

## Abstract

The development of a computerized database on advanced structural ceramics can play a critical role in fostering the widespread use of ceramics in industry and in advanced technologies. A computerized database may be the most effective means of accelerating technology development by enabling new materials to be incorporated into designs far more rapidly than would have been possible with traditional information transfer processes. Faster, more efficient access to critical data is the basis for creating this technological advantage. Further, a computerized database provides the means for a more consistent treatment of data, greater quality control and product reliability, and improved continuity of research and development programs.

A preliminary system has been completed as phase one of an ongoing program to establish the Structural Ceramics Database system. The system is designed to be used on personal computers. Developed in a modular design, the preliminary system is focused on the thermal properties of monolithic ceramics. The initial modules consist of materials specification, thermal expansion, thermal conductivity, thermal diffusivity, specific heat, thermal shock resistance, and a bibliography of data references. Query and output programs also have been developed for use with these modules. The latter program elements, along with the database modules, will be subjected to several stages of testing and refinement in the second phase of this effort. The goal of the refinement process will be the establishment of this system as a user-friendly prototype.

Three primary considerations provide the guidelines to the system’s development: (1) The user’s needs; (2) The nature of materials properties; and (3) The requirements of the programming language. The present report discusses the manner and rationale by which each of these considerations leads to specific features in the design of the system.

## Introduction

Technical advances in materials research are occurring at a rapid pace in all aspects of the development and refinement of advanced ceramics. As a result, technical data are proliferating at an exponentially increasing rate. Industries may reasonably anticipate new technological opportunities to accompany these advances in materials research. However, some of these opportunities will be lost if the new data are not successfully communicated to the design engineers who can transform the data into new or better products. The traditional communication routes for disseminating technical data frequently have slow diffusion rates and may be cumbersome to use, especially when disciplinary boundaries are crossed. To capture the data and to fashion it into an expeditiously useful form, advances in computerized information systems are being pursued vigorously in a wide range of scientific and engineering fields [1–7], and involve national agencies [8], professional societies [9], and dedicated nonprofit organizations [10]. The present report discusses the initial results of the effort at the National Institute of Standards and Technology to develop a new database system that will be focused on critical material properties of advanced structural ceramics. The current effort is being conducted in conjunction with the research program at the Center for Advanced Materials established at the Pennsylvania State University by the Gas Research Institute.

The rapidly increasing importance of computerized information systems is readily apparent to even the most casual observer. The number of information sources in technical areas is enormous. The subject matters within these areas are increasingly diversified. There are a steadily proliferating number of specializations within each subject area. Each specialization generates technical terminology not in common with other specializations. And, the objectives for obtaining, developing, or using the data are unlimited.

Coping with this abundance and diversity of information is the function of the computerized data-base. By using the logistical power of computers, data can be stored, sorted, searched, retrieved, and used in a very small fraction of the time required by manual methods. Further, data stored in a computerized database may form the basis of a technical “corporate memory” because the availability and usefulness of the data persist beyond the lifetime of the project that generated the data. As a result, there is not only a more rapid utilization of technical advances, but also a reduction of wasteful duplication of efforts. This powerful processing of information can create improved perceptions of research strengths and weaknesses and, hence, may provide improved managerial vision for future research planning.

The Structural Ceramics Database (SCD) system is being developed so that these capabilities can be used to help industry in taking advantage of newly emerging specialized ceramics. This objective has two inherent requirements. The system must include critical data, and the system must be easy to use. The former requirement pertains to the content of the database. The latter requirement pertains to how the computerized system is constructed. The SCD project, therefore, has both a data acquisition component and a system development activity.

The first phase of the SCD project was focused on the development of a preliminary software system. The initial emphasis, therefore, was on technical considerations, and the result was a preliminary system that has fully functioning storage, search, and retrieval capabilities.

The first step of the project was to consider the needs of the user. The user’s requirements formed the basis for specifying the technical requirements of the underlying database management system (DBMS) that would be selected as the programming language. To further guide the development of the system, a specific application area, high temperature gas-fueled heat exchangers, was selected, and the gathering of data was started. These factors were combined in determining the initial structure of the database system that will be discussed in the following sections.

## Issues

The development of the SCD system requires the resolution of three sets of issues: the design issues that result from consideration of the user’s needs [11–12], the technical issues that result from the constraints of the programming language [13–14], and the materials properties issues that result from the nature of the materials and the particular properties that are to be included in the database.

### The User Interface

First and foremost, the system must be easy to use. The system should place very little demand upon the user in terms of technical knowledge of computers or computer programming. Once started, the system should guide the user at each step of the interactive session, always clearly indicating the user’s options. Never should the user need to refer to an operations manual.

At the same time, the intelligence of the user must be respected. The user of a database wants to extract information that usually has a well defined scope and content. The system must make it easy for the user to pose questions to the database in a clear and concise manner, and the answers to the questions must be presented to the user quickly and in a readily understood format. Those answers should also be expressed in units with appropriate dimensions and terminology that are convenient and comfortable for the user. The answers must also be comprehensive in the sense that the user’s ranges of conditions and questions are anticipated, so that the questions *can* be asked, and that there are data available to answer them. Further, whenever the user wants additional information regarding any specific data, the system should provide references to readily available literature.

To accomplish these features, the design of the database should include three primary characteristics: simple screens; menus with light-bars; and on-line help.

In many respects, what appears on the screen is the immediate link between the database and the user. The appearance of the screen, therefore, should not assault the user’s senses but, rather, should focus the user’s attention on the central concern. Too much information presented to the user on the screen creates confusion and distraction. To make a screen simple, the program sometimes must bear the responsibility for considerable preparatory steps prior to presenting the principal screen. As a result, the design of a simple screen is often, ironically, more difficult than the design of a complicated screen.

The use of a menu is probably the most important means of simplifying the interaction between the database and the user. When menus are used, there is no need or requirement for the user to remember program commands that are too frequently crytic, obscure, or unclear. The options are always presented to the user who only needs to select one of them. As a result, there are fewer entry errors. Further, the database system is always in control of the program flow, i.e., the user can only ask the database to do operations that it is ready to do. If primitive DBMS commands are accessed directly, it is possible to ask a program to execute operations before all of the preparatory steps have been completed. In this situation, the program can “hang” or “crash”. With a menu-driven system, the responsibility for maintaining the proper program flow rests with the design of the system.

Menu systems are further enhanced by the use of light-bars, a rectangular area that appears on the screen as a highlighted region. The light-bar highlights one option and can be moved by the user to any other option. To make a selection from among the options, the user merely moves the light-bar to highlight the desired option and presses the return key. Exactly the same set of keystrokes are used with every such menu. Hence, the user’s need to be familiar with the mechanical aspects of the keyboard are minimized. Light-bars can also be programmed so that a pointing device such as a mouse can be used, thereby entirely eliminating keystrokes for menu selections. Also important is the fact that the user *sees* what the choice is when the light-bar highlights an option.

The information and the options presented to the user of a materials property database must relate to the technical content of the database. Consequently, the words and terminology used on the screen must be tailored to the technical material. Technical terminology is rarely standardized across all disciplines. Therefore, it is essential that online help be available to the user at any time to explain the options and the terminology, and to provide references to the literature where an indepth discussion on the subject matter can be found. Online help provides information to the user immediately, when it means the most to the user.

### Materials Property Issues

The design of a database necessarily requires consideration of the information to be contained in the database. Data in a database occurs in four types: numeric (numbers), character (words), logical (true or false), and date (month, day, and year). Before the database can be prescribed, the type of data that it is to contain must be known. The specification of what specific information is needed for a materials property database is less obvious than it might seem at first thought. For example, consider what is required to identify a particular structural ceramic material. What characteristics uniquely define the material? Conversely, can the material be grouped with other materials as part of a more general class?

It is widely recognized that the name of a ceramic material does not provide an adequate description of the material. For example, there are many forms of silicon nitride. All silicon nitrides have the same primary chemical formula, Si3N4, but the sintering aids, impurity components, porosity, and microstructure are different. This question of identifying a ceramic material is being investigated currently by the ASTM Committee E-49, Computerization of Material Property Data. Their deliberations indicate that at least 10 categories of supporting information, also called metadata, table 1, are desirable for the specification of an advanced material [15]. These descriptors only identify the material and do not provide any of the material properties.

Each material property to be included in the database also must be given carefully selected specification data. What is the property? What method was used to measure it? Are the data of reasonable quality?

The range of properties that should be included in the database depends on the application or the intended use of the database. A general database that is not focused on any particular application would need to include far too many properties than would be practical. Rather than construct a data-base that is entirely comprehensive, it is preferable that the database be developed for a well focused application for which the critical data can be clearly determined.

For any given property, an important issue pertaining to the usefulness of the data is how much supporting detail should be available to describe how the property value was determined. What depth of information is required concerning the method used, the conditions of the experiments, or the statistical treatments that may have been applied to the data? These questions are encountered whenever data are reported in the technical literature. Technical papers are usually required to provide in some manner a complete description of all experimental apparatus, experimental procedures, and data analysis. A database is not intended, and should not be expected, to replace a technical paper. However, it may be important to know what experimental techniques were used to evaluate the property. Different methods may subject the material to different conditions, and hence, the results from one technique may be more appropriate to the user’s application than other techniques. To accommodate the need to be clear, succinct, and complete with respect to property data and its determination, a bibliography of technical references should be maintained as part of the metadata used to describe and record material properties.

The final concern regarding the data is the quality of the values recorded in the database. Some assessment of the quality of the data, its accuracy or reliability must be provided with the value that is recorded in the database. If there are limitations on the validity of the value, the user needs to be forewarned of the limitations. The design of the database, therefore, should include metadata fields in which the quality and limitations of the data can be noted.

### Constraints of the Programming Environment

The programming environment for the development of a database is most conveniently and wisely taken to be a commercially available database management system (DBMS). A DBMS is essentially a language that can be used to tell the computer how to store and retrieve data. Commercial DBMS packages contain many highly refined features that greatly facilitate the creation of a database architecture and provide the essential means to search for information stored within the resulting structure. These features, while sophisticated and desirable, may also be viewed as constraints on the database design. Thus, it is important to identify the technical features that are necessary to secure compatibility with the data that are to be included in the database and to ensure the fulfillment of the requirements of the user.

The SCD must consist of many material properties and characteristics, including the materials specification, chemical composition, microstructure, mechanical properties, and thermal characteristics. Each piece of information that is to be included in the database must be alloted a distinct amount of space where the information can be stored. In the simplest structure of a database, the pieces, called fields, are concatenated to form a single collection of information, called a record,

**Figure:**
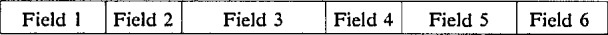

as illustrated in the diagram. In this example, six fields of different sizes are joined together on one line to form one record. The complete database may then be visualized as a collection of several such records, one per line, with each field aligned to form a column. A collection of three records would look like the following matrix:

**Figure:**
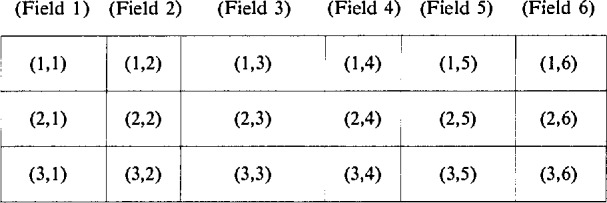

In principle, this record structure can be used for any database. In practice, however, this structure can be somewhat awkward and inconvenient.

To illustrate this point, consider a simple data-base that contains only selected thermal properties of materials as a function of temperature. For the purpose of discussion, assume that the fields for this hypothetical database are restricted to the name of the material, the temperature, the thermal conductivity, and the thermal shock resistance. Suppose also that results for alumina have been obtained from a published source that reported the thermal conductivity at three temperatures. Then, the data-base with only this data would be:

**Figure:**
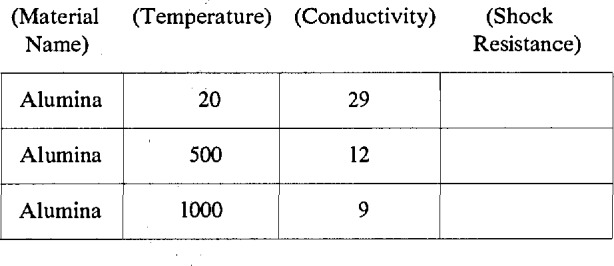

The database has three records corresponding to the three temperatures at which the thermal conductivity was determined. The field containing the Material Name has the same information in each record. This redundancy of information is typical of much of the supporting information that is required to make the database useful. Many more fields with such metadata would be necessary to complete the specification of a good database on thermal conductivity, including the units of temperature and thermal conductivity, detailed information on the composition and microstructure of the material and the processing technique used to make the material, and details about the measurement method. The proliferation of such fields can rapidly lead to a high degree of redundant information in the database. In the example, the field for Shock Resistance is not only redundant, but also superfluous since no values for that property were given in the referenced report.

Redundant and superfluous data are wasteful of limited storage space, reduce the speed with which the information can be processed, and increase the potential for errors in the entry of the information into the database. To avoid this situation, a different type of database structure is needed. Instead of linking all the fields together into a single record, the fields can be divided into logical subgroups. Either a hierarchial or a relational database structure can accomplish this organization.

Hierarchial databases achieve a logical organization and space efficiency by creating a tree structure. Each new component to the database becomes a new branch in the tree. If a component is not used, that branch does not need to be created. Thus, the hierarchial system does not require any wasted space. However, maintenance or revision of the tree structure can be cumbersome, and navigating the tree from one record to another can be awkward.

Relational databases provide a more desirable structure for scientific and engineering applications that may anticipate a need for revision as the discipline progresses. To illustrate this point, consider the preceding example. The original database could be divided into three component databases, one each for materials specification, thermal conductivity, and thermal shock resistance. In a relational database system, each of these components can be maintained independently, provided that a unique relation between the components is specified and preserved. This relationship can be established, for example, by adding a new field, called a key, that identifies the source of the data in each component. In the example, the three subgroup databases could be defined as follows:

**Figure:**
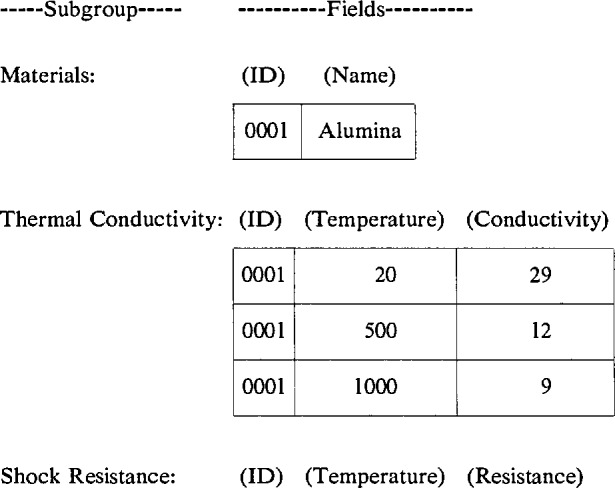

The greater efficiency of the relational database structure is readily apparent even in this restricted example. The addition of the ID field is sufficient to make the relationship between the subgroups clear and unambiguous. The new structure completely eliminates the previously redundant and superfluous data. For a materials property database, a fully developed subgroup might contain as many as 50 fields. Hence, the relational database structure may be considered the preferred structure for scientific and engineering databases.

In the example, it may be noted, further, that three records were required to record the thermal conductivity at three temperatures, even though all three results were obtained from one reference. Logically, it would be easier to search and retrieve this information if all the values were contained in one record. Advanced DBMS packages provide this data structure in the form of multiple entry associated fields. In a multiple entry field, a variable number of different values can be entered. Associated fields are multiple entry fields for which there is a one-to-one correspondence between the associated entries. For example, if temperature and conductivity are associated fields, then the Thermal Conductivity subgroup described above would become:

**Figure:**
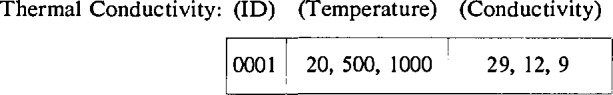

The first entry in the temperature field, 20, corresponds to the first entry in the conductivity field, 29. Associated fields capture the entire set of relevant data in the single record, thereby making the search and retrieval operations more efficient and faster. The entire example database, recast as a relational database with associated, multiple entry fields, is reduced to only the following:

**Figure:**
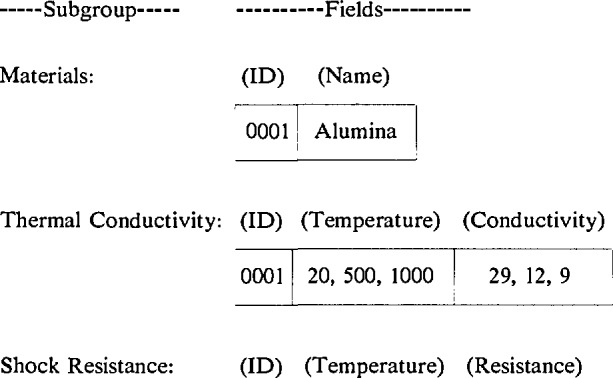

Most materials property information occurs with parametric dependencies and with many metadata fields. Hence, associated multiple entry fields in a relational database are natural and desirable refinements of the database architecture for scientific and engineering applications.

Next to the specification of the underlying data-base structure, the most important consideration for the programming environment is the ability to search the database for information. Indeed, it is this stability that makes databases useful and powerful. As a result, the search and retrieval features are the highlights of many of the advanced DBMS packages available commercially. In making comparisons of search and retrieval capabilities, it is important to recognize that most searches are specified in terms of the supporting metadata rather than the property itself. Many of the meta-data fields are textual in character, i.e., collections of words. To find what the user has specified, the DBMS may need to search *within* a field to find the user’s words embedded within the field. Not all DBMS packages permit this type of search. For materials property databases, searching within a field may be essential for characteristics such as chemical composition, microstructure, or processing conditions. A summary of the general characteristics that may reasonably be expected of a DBMS for scientific and engineering applications is given in table 2.

## Structural Ceramics Database

The development of the SCD system has been structured in a logical sequence of steps. Initially, a particular application was selected to provide a definite focus for the choice of properties to be included in the database. The particular application was selected after conducting a survey of the activities of the project’s founding sponsor, the Gas Research Institute (GRI) [16]. GRI contractors indicated that tailoring the system for use with heat exchanger and recuperator design would be especially valuable to them in both the short-term and the long-term. Further discussions with GRI contractors and other research and industrial contacts produced a list of critical properties, i.e., those properties that are needed for the design efforts and for which current information is often inaccessible, cumbersome to find, and/or essential to the design. With a focused application and a sampling of the typical data to be included in the database, the general technical specifications were formulated. The latter specifications were coupled to the system needs, as seen from the user’s point of view, to determine the overall requirements for the basic database management system (DBMS), as summarized in table 2. Based on those requirements, a commercially available DBMS was selected.

The system software design effort was then aimed at developing a fully functioning prototype system. The construction of the prototype was begun using a modular design consisting of general modules that may be used for any target application, and tailored modules that pertain only to specific material properties.

The modular design provides considerable flexibility and allows the system to be expanded or adapted to diverse applications. This design is also being exploited in a pragmatic way. It is perhaps well known that it is much easier to develop a programmer’s working system than it is to develop a working *user-friendly* system. It may also be readily understood that certain data entry and review modules are necessary before data can be entered into the system and be verified. Consequently, to maximize the efficiency of the system development, phase one of this project was directed towards the development of those modules that would provide a working, preliminary system as soon as possible. The user interface modules are scheduled to be developed in the second phase of the project.

The preliminary system of the phase one effort is now complete. The preliminary system consists of modules for materials specification, thermal expansion, thermal conductivity, thermal diffusivity, specific heat, thermal shock resistance, a bibliography of data references, queries, and output. Currently, the query and output modules are rather general, for development purposes, and need to be streamlined before being used in the prototype of phase two. The other modules are ready for testing in their current forms.

Tables 3–9 summarize the contents of the current primary information modules. For some fields, such as Material Class, the entries are restricted to a small, finite, closed set. The possible entries for those fields are also shown in the tables.

The initial materials specification module has been constructed in accordance with the guidelines evolving from the deliberations of ASTM Committee E-49, Computerization of Material Property Data. The guidelines, summarized in table 1, have been implemented by dividing the required information into 32 fields. In brief, these fields describe what the material is called, how and where it was made, and what physical and chemical characterization information has been recorded.

The materials property databases are constructed with the sets of information fields intentionally kept small. The user’s first priority is to know the value of a property under a specifed condition. In general, each of the measurement methods has certain measurement conditions associated with it. Thus, identifying the method effectively identifies the conditions. If complete details of a particular measurement are needed, the user may consult the literature reference that is included in the bibliographic database. Further, a help function provides general references in which descriptions of all the related measurement techniques may be found.

## Discussion

From the point of view of the user, the basic function of the database is to help the user obtain information. To do this, the database system must have a flexible, but user friendly, query capability. The essence of the query system in the SCD is illustrated in the following flow chart:

**Figure:**
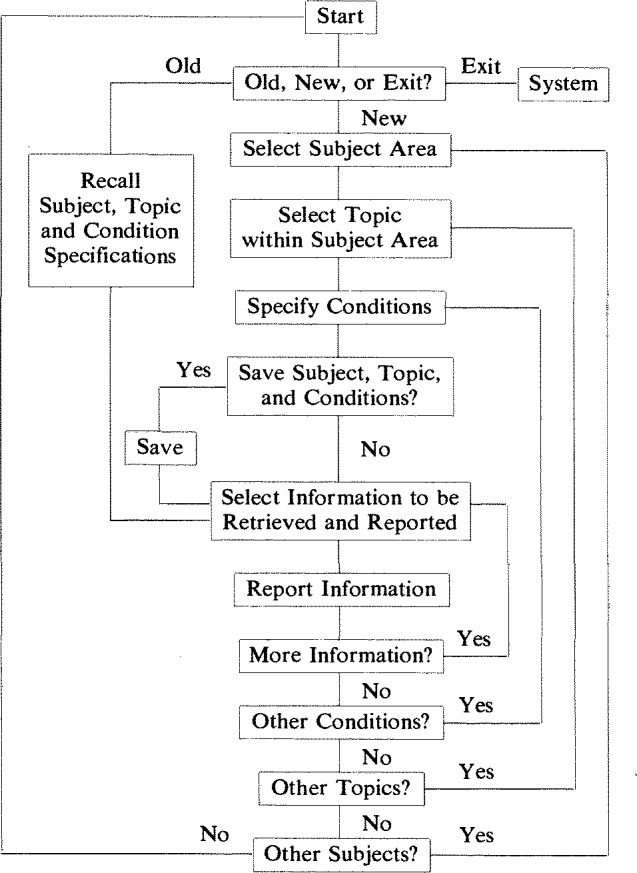

Any query session may be saved for future use. Therefore, at the beginning of a new session, the user is given the opportunity to recall a previous query or to start a new one. A new investigation begins with the selection of the central subject on which the search of the SCD will be based. The subject could be a material, a property, or a bibliographic reference. For example, to examine the properties of a silicon nitride, the subject would be ceramic materials and the topic would be silicon nitride. Further specifications, such as sintering aids, phase composition, or a maximum value for the thermal expansion coefficient, could be made in the Specify Conditions step. When the user is satisfied with the constructed query, the query conditions may be saved for future reference.

The SCD system uses the query conditions to select from the entire database only the subset of records that fulfill the user’s requirements. The user may then choose to examine any part of the information in the subset. For example, having specified silicon nitride with MgO as a sintering aid and a linear thermal expansion coefficient not greater than 3.5 × 10^−6^ K^−1^, the user could readily determine the variation of the thermal shock resistance with respect to fabrication process. The latter information would be obtained by specifying that the thermal shock resistance and the fabrication process be included in the list of items reported to the user.

The SCD query program is more powerful than the simplified flow chart reveals. Indeed, the program currently is too powerful to be user friendly. Within the specifications of conditions, it is possible to conduct several independent queries and then combine them into a single complex query. However, complex queries require the user to have a considerable knowledge about the structure and content of the fields in the various databases. An objective of the user interface module will be to harness the power of the query program so that the user can find precisely the information that is wanted, without an in-depth knowledge of the SCD system.

## Conclusion

The Structural Ceramics Database (SCD) system is being developed as a means of accelerating advances in ceramics-based technology. The first phase of the ongoing SCD program has resulted in a preliminary system for use with personal computers. This phase-one system is focused on the thermal properties of monolithic ceramics. The modular design of the system permits independent modules for materials specification, thermal expansion, thermal conductivity, thermal diffusivity, specific heat, thermal shock resistance, and a bibliography of data references. Accessing the information contained in these modules is accomplished by query and output program elements.

The design of the system has been based on an analysis of three primary considerations relating to the user, the materials properties, and the programming language. Each consideration imposes constraints on the design of the system. The user’s interest is the principal determinant of the content of the database and the design of the manner and style with which the system interacts with the user. The programming language determines the technical limitations on how the data are actually managed. The materials properties determine what provisions are necessary to ensure that the critical information is adequately and accurately communicated to the user. The latter provisions impose constraints, for example, on data validation routines for data entry and on query structures for data retrieval.

The success of the SCD system will rest first on its emphasis on user-friendliness and second on its content of critically important data. The convenience, speed, and efficiency of the access to the data will enable developments in research to be transferred to industrial applications far more rapidly than could be expected with the traditional technology transfer processes.

## Figures and Tables

**Table 1:** Preliminary guidelines from ASTM Committee E-49 regarding the categories of information needed to describe a material for database purposes

Material Class
Specific Material within a Class
Material Designation
Material Condition
Material Specification
Producer or Source of Material
Producer Lot Number and/or Assigned Reference Number
Product Form
Material Composition
Fabrication History

**Table 2:** Essential requirements for a database management system (DBMS) applied to scientific and engineering information systems

Relational database structure
Variable length fields
Large field lengths, in kilobyte range
Multiple entry fields
Associated fields
Large number of fields per record
Large total record sizes, in kilobyte range
Very large maximum number of records
High efficiency indexing
Concurrent indexing
Multiple field keys for indexing
Search on any field
Search within a field
Logically concatenated searches on several fields
Compatibility with other computer languages
Compiled or runtime codes

**Table 3:** A listing of the information fields contained in the materials specification module. Where only fixed entries are allowed, the alternatives are listed below the field topic

Material class:
Monolithic ceramic
Structure class:
Polycrystalline, Single crystal, Graphitic, Amorphous
Chemical class:
Carbide, Nitride, Oxide
Chemical name
Chemical Abstract Service Number
Chemical formula
Source of the material
Manufacturer’s designation for the material
Manufacturer’s lot number
Product date
Standard design specification code
Organization setting standard specification
Supplementary information regarding specification
Fabrication process:
Slip casting. Tape casting, Sintering (firing), Extension,
Mechanical throwing, Die pressing, Isostatic pressing,
Injection molding, Hot pressing, Glass ceramics route,
Glazing, Plasma spray, Chemical vapor deposition,
Crushed ground
Fabrication form;
Plate, Bar, Rod, Wire, Tube, Thick film, Thin film, Powder
Fabrication history
Specimen state: Virgin, Modified
Description of specimen modification
Location of the specimen from within the fabrication form
Elemental composition of the material
Weight percents of the elemental components
Standard deviations of weight percents of elemental components
Phase composition of the material
Weight percents of the phase components
Standard deviations of weight percents of the phase components
Mean value of the bulk density of the material
Unit of density: g/cm’, kg/m’
Theoretic density
Grain size
Unit of grain size: jam, mm, m
Known or intended applications of this material
Supplementary notes

**Table 4:** A listing of the information fields contained in the thermal expansion module. Where only fixed entries are allowed, the alternatives are listed below the field topic

Measurement method:
Push-rod dilatometer, Twin tele-microscope, Interferometer,
Single crystal x-ray diffraction. Powder x-ray diffraction,
Neutron diffraction, High speed pulsed heating,
Volumetric dilatometry, ASTM C372 (Dilatometry),
ASTM E289 (Interferometry),
ASTM E831 (Thermodilatometry), Other
Sample preparation/pretreatment
Measurement notes
Quality
Cautions
Unit of temperature: °C, °F, K
Unit of thermal expansion coefficient: 10^−6^ K^−1^
Thermal expansion coefficients at temperature:
Temperature
Bulk average value
Value along *a*-axis
Value along *b*-axis
Value along *c*-axis
Polynomial representation of principal axial coefficients α(i, i):
Temperature range from ____ to ____
α(1,1)=____+____(*T*/1000)+____(*T*/1000)^2^
α(2,2)=____+____(*T*/1000)+____(*T*/1000)^2^
α(3,3)=____+____(*T*/1000)+____(*T*/1000)^2^

**Table 5:** A listing of the information fields contained in the thermal conductivity module. Where only fixed entries are allowed, the alternatives are listed below the field topic

Measurement Method:
Longitudinal heat flow, Forbes’ bar, Radial heat flow,
Direct electrical heating, Thermoelectric,
Thermal comparator, Periodic heat flow, Transient heat flow,
ASTM C201 (Comparative), Other
Sample Preparation/Pretreatment
Measurement Notes
Quality
Cautions
Unit of temperature: °C, °F, K
Unit of thermal conductivity: W m^−1^ K^−1^
Temperature
Thermal conductivity

**Table 6:** A listing of the information fields contained in the thermal diffusivity module. Where only fixed entries are allowed, the alternatives are listed below the field topic

Measurement Method:
Center-heated long bar, End-heated long bar,
Moving heat source, Small area contact,
Thermoelectric effect, Semi-infinite plate, Radial heat flow,
High intensity are, Flash heating, Electrically heated rod,
Angstrom’s method, Modified Angstrom’s method,
Temperature wave velocity,
Temperature wave amplitude-decrement, Phase lag,
Radial wave, ASTM C351 (Insulating Materials),
ASTM C714 (Thermal Pulse), Other
Sample Preparation/Pretreatment
Measurement Notes
Quality
Cautions
Unit of temperature: °C, °F, K
Unit of thermal diffusivity: m^2^ s^−1^
Temperature
Thermal Diffusivity

**Table 7:** A listing of the information fields contained in the specific heat module. Where only fixed entries are allowed, the alternatives are listed below the field topic

Measurement Method:
Nernst-type adiabatic vacuum calorimeter,
Modified adiabatic calorimeter, Drop ice calorimeter,
Drop isothermal water calorimeter,
Drop copper block calorimeter, Pulse heating,
Comparative method, ASTM C351 (Insulating Materials),
Other
Sample Preparation/Pretreatment
Measurement Notes
Quality
Cautions
Unit of temperature: °C, °F, K
Unit of specific heat: J kg^−1^ K^−1^
Temperature
Specific heat

**Table 8:** A listing of the information fields contained in the thermal shock module. Where only fixed entries are allowed, the alternatives are listed below the field topic

Measurement Method:
Water Quench/Internal Friction, Other
Sample Preparation/Pretreatment
Measurement Notes
Quality
Cautions
Unit of temperature: °C, °F, K.
Critical quench temperature difference

**Table 9:** A listing of the information fields contained in the bibliography module. Where only fixed entries are allowed, the alternatives are listed below the field topic

Names of authors
Total number of authors
Country of authors
Title of paper/report/article
Type of publication:
Journal, Book, General report, Contract report, Conference, Dissertation/Thesis, Patent, Magnetic tape, Private, Other
Name of publication medium
Names of editors of the publication medium
Language of publication
Chapter number
Volume number
Issue number
Page numbers
Publication date
Publisher
Sponsor
Site of conference, meeting, university granting degree, or other site
Patent number
Patent country
Initial source of abstract citation
Chemical Abstract Service abstract number
International Standard Serial Number
Number of materials discussed
Names of materials
Synonyms for materials
Chemical Abstract Registry number
Physical properties discussed:
Lattice parameter
Chemical properties discussed:
Corrosion, Corrosion products, Corrosion rate, Oxidation, Chemical reactivity, Stability
Thermal properties discussed:
Thermal expansion, Thermal conductivity,
Thermal diffusivity, Specific heat, Thermal shock,
Thermal emissivity
Mechanical properties discussed:
Elastic modulus, Elastic constants, Young’s modulus (*E*),
Shear modulus, Poisson’s ratio, Bulk modulus,
Compressibility, Strength, Tensile strength, Flexure strength,
Bend strength, Fracture strength, Rupture strength,
Stress-strain, Fracture energy, Fracture toughness,
Toughness, R-curve, Critical stress intensity factor,
Crack growth, Creep, Creep rupture, Fatigue, Cyclic fatigue
